# Control of Inflammatory Response by Tissue Microenvironment

**DOI:** 10.1101/2024.05.10.592432

**Published:** 2024-05-14

**Authors:** Zhongyang Wu, Scott D. Pope, Nasiha S. Ahmed, Diana L. Leung, Stephanie Hajjar, Qiuyu Yue, Diya M. Anand, Elizabeth B. Kopp, Daniel Okin, Weiyi Ma, Jonathan C. Kagan, Diana C. Hargreaves, Ruslan Medzhitov, Xu Zhou

**Affiliations:** 1 Division of Gastroenterology, Hepatology and Nutrition, Department of Pediatrics, Boston Children’s Hospital and Harvard Medical School, Boston, Massachusetts 02115, USA; 2 Broad Institute of Massachusetts Institute of Technology and Harvard, Cambridge, Massachusetts 02142, USA.; 3 Department of Immunobiology, Yale University School of Medicine, New Haven, Connecticut 06510, USA; 4 Howard Hughes Medical Institute, Yale University School of Medicine, New Haven, Connecticut 06510, USA; 5 Molecular and Cell Biology Laboratory, Salk Institute for Biological Studies, La Jolla, CA, USA.; 6 Ministry of Education Key Laboratory of Cell Proliferation and Differentiation, School of Life Sciences, Peking University, Beijing 100871, China; 7 Division of Pulmonary and Critical Care Medicine, Massachusetts General Hospital, Boston, Massachusetts, 02115; 8 Tananbaum Center for Theoretical and Analytical Human Biology, Yale University School of Medicine

## Abstract

Inflammation is an essential defense response but operates at the cost of normal functions. Whether and how the negative impact of inflammation is monitored remains largely unknown. Acidification of the tissue microenvironment is associated with inflammation. Here we investigated whether macrophages sense tissue acidification to adjust inflammatory responses. We found that acidic pH restructured the inflammatory response of macrophages in a gene-specific manner. We identified mammalian BRD4 as a novel intracellular pH sensor. Acidic pH disrupts the transcription condensates containing BRD4 and MED1, via histidine-enriched intrinsically disordered regions. Crucially, decrease in macrophage intracellular pH is necessary and sufficient to regulate transcriptional condensates *in vitro* and *in vivo*, acting as negative feedback to regulate the inflammatory response. Collectively, these findings uncovered a pH-dependent switch in transcriptional condensates that enables environmental sensing to directly control inflammation, with a broader implication for calibrating the magnitude and quality of inflammation by the inflammatory cost.

## Introduction

Inflammation is crucial for maintaining homeostasis and defending the integrity of tissues and organs. Yet, excessive inflammatory responses can result in significant tissue damage.^1^ Much of our knowledge in the protective and pathological roles of inflammation relates to how inflammatory responses impact organ functions. By contrast, less is known about how the state of a given tissue impacts the inflammatory response. The magnitude, duration and specific impact of inflammatory response must be calibrated to both the presence of inflammatory triggers and the extent of pathological outcome.^2^

One universal feature of tissue homeostasis is the maintenance of a stable pH range. Different tissues and organs maintain an interstitial environment within specific homeostatic pH range, whereas these pH levels are often perturbed during inflammation.^3^ For instance, blood pH is tightly regulated between 7.35 and 7.45 through respiration and renal compensation.^4^ In patients with sepsis, severe acidosis often indicates a poor prognosis for survival.^5,6^ In the brain, the cerebrospinal fluid maintains a mildly acidic pH ~7.3; however, ischemic injury can lower this to pH 6.6. The pH of the paracortical zone of lymph nodes is sustained between 6.3 and 7.1, but becomes even more acidic upon infections.^7^ Solid tumors frequently exhibit acidic pH due to heightened metabolic activity, hypoxia, and active proton extrusion by cancer cells.^8^ These deviations from normal pH levels can indicate an emergent state in affected tissues.

In mammalian cells, pH levels can be monitored both extracellularly and intracellularly. On the cell surface, a broad range of pH values can be detected by a variety of extracellular sensors, including G-protein coupled receptors (GPR4, GPR65, GPR68), acid sensing ion channels (ASIC1, ASIC2, ASIC3, ASIC4) and transient receptor potential cation channel subfamily V member 1 (TRPV1).^4^ These extracellular pH sensors are activated at the pH range of 4 to 8,^9,10^ regulating various cellular and physiological parameters, from blood pH and lipid metabolism to pain sensations during exercise.^11–13^ In contrast, the mechanisms intracellular pH sensing and the subsequent cellular responses are less understood. The Hypoxia-Induced Factors, HIF1 alpha and HIF2 alpha, can be activated by acidic conditions independently of hypoxia.^14,15^ In the context of cancers, transcription factors SMAD5 and Sterol Regulatory Element-Binding Protein 2 (SREBP2) have been implicated in cellular responses to intracellular pH in cancer cell lines.^16,17^ It remains to be determined whether and how pH-sensing mechanisms specifically regulate cellular activities in the context of inflammation. Despite this lack of understanding, acidic pH is generally considered suppressive to cell activation and proliferation. Change in pH may influences survival, differentiation, migration and cellular metabolism in a cell-type specific manner.^3^

In considering immune cells that may sense changes in pH and influence tissue activity, we focused on macrophages given their role as tissue sentinels.^18,19^ In response to cues associated with microbial infections, macrophages regulate the expression of thousands of genes important for inflammation, host defense, antiviral response, coordination with adaptive immune system and tissue homeostasis.^20–23^ Recent studies revealed that proteins can form biomolecular condensates of fundamental roles in cellular organization, signaling, stress response and gene regulation.^24–29^ In particular, critical factors involved in transcription tend to concentrate in distinct and dynamic nuclear foci referred to as transcription “hubs” or “condensates”. These condensates represent crucial biochemical controls of gene expression, transcription burst and compartmentalization of opposing regulators.^30–35^ The proteins identified within these foci, including BRD4, MED1, Pol II, YAP/TAZ, TBP, P300, pTEFb, typically contain intrinsically disordered regions (IDRs) that favor weak, multivalent interactions.^34–41^ Specifically, BRD4 is a member of the bromodomain and extraterminal (BET) protein family that recognizes histone lysine acetylation associated with active transcription.^42,43^ It interacts and recruits the mediator complex, pTEFb and other transcription machinery, as an essential regulator of Pol II-dependent transcription.^44,45^ In macrophages and in mice, inhibition of BRD4 reduces inflammatory response and prevents lethality in severe sepsis,^46^ suggesting that the function of BRD4 is vital for the activation of inflammatory response. Despite the increasing examples of transcriptional condensates in gene regulation, regulation of transcriptional condensate formation and their roles in the immune system are not well understood.

**Figure 1.**
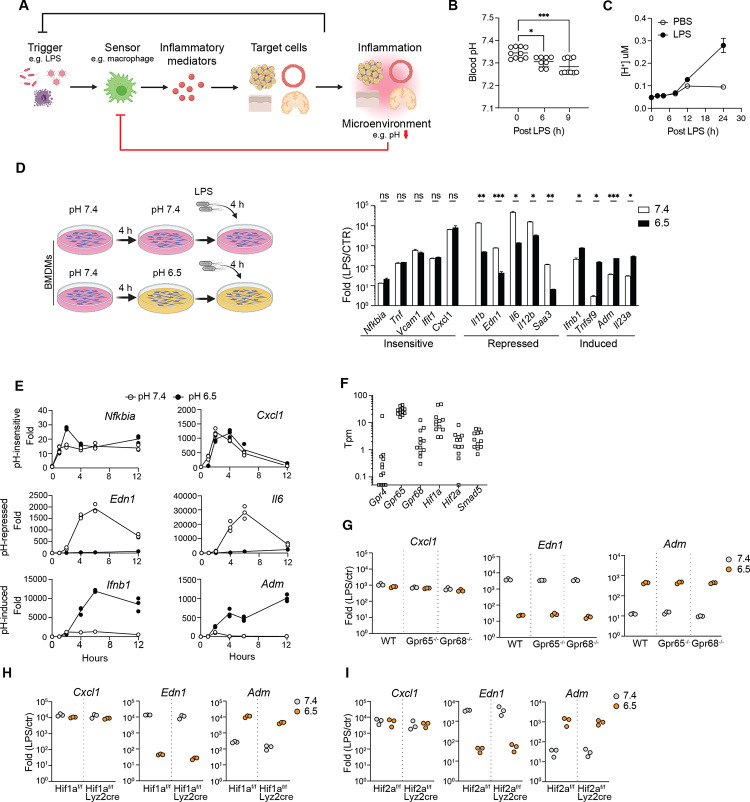
Acidic pH regulates gene-specific inflammatory response in macrophages. (**A**) A diagram of an inflammatory circuit with pH-sensing feedback to the innate sensors. (**B**) Blood pH in mice challenged with 10 mg/kg LPS intraperitoneally. One-way ANOVA, Dunnett test for multiple comparisons, Mean +/− STD. (**C**) Extracellular acidification of BMDMs stimulated with 100 ng/mL LPS *in vitro*. Mean+/− STD. (**D**) Fold activation of inflammatory genes in BMDMs after 4 hours 10 ng/mL LPS at pH 7.4 or 6.5, normalized to unstimulated conditions respectively. Mean+/− standard deviation (STD). Unpaired t-test, Holm-Sidak’s test for multiple comparisons. (**E**) Time course analysis of gene activation in BMDMs stimulated with 10 ng/mL LPS at pH 7.4 and 6.5. (**F**) Expression of known pH sensors in macrophages, including BMDMs, monocytes, tissue resident macrophages in peritoneal, liver, lung, small and large intestines (ref: Lavin 2014). (**G-I**) Fold changes of selected genes in WT, *Gpr65*−/− and *Gpr68*−/− BMDMs (G), *Hif1a*−/− BMDMs (H) and *Hif2a*−/− BMDMs (I). In (B) and (D), *p<0.05, **p<0.01, ***p<0.001.

In this study, we explored the hypothesis that macrophages use the detection of intracellular pH deviation as a guide to control inflammatory responses (Figure 1A). Using the Toll-like receptor 4 (TLR4) ligand bacterial lipopolysaccharide (LPS), we found that changes in pH do not impact TLR4 signal transduction per se, but strongly impact the spectrum and the extend of inflammatory genes activated by TLR4. We identified BRD4 as a novel intracellular pH sensor. The BRD4-containing transcription condensates are pH-sensitive, regulated by the evolutionary conserved histidine-enriched IDR. The inflammatory activation of macrophages triggers intracellular acidification and alters transcriptional condensates *in vitro* and *in vivo*. Thus, this work reveals a new regulatory mechanism of inflammation, where transcriptional condensates integrate extracellular and intracellular pH via BRD4 to elicit gene-specific inflammatory response based on microenvironment. Sensing pH deviation by the immune system may act as a feedback mechanism to balance the protective benefits of immune response against their potentially pathological impact.

## Results

### Acidic pH modulates the gene-specific inflammatory response

In a murine model of acute inflammation, intraperitoneal (i.p.) injection of a sub-lethal dose of LPS in wild-type mice (WT) triggered a TLR4-dependent systemic inflammatory response. As a consequence, the blood pH decreased significantly at 6 hours post-injection (Figure 1B). Severe acidosis (< pH 6.5) was reported in various tissues at 24 hours, including liver, small intestine, kidney, brain stem, hypothalamus and cortex of the brain,^47^ underscoring the link between acidic pH levels and acute inflammatory response. *In vitro*, WT bone-marrow derived macrophages (BMDMs) acidified the extracellular environment to pH 6.5 at 24 hours after LPS stimulation (Figure 1C and S1A), in the presence of a physiological level buffer system (23.8 mM bicarbonate). Notably, this pH level did not adversely affect the viability of BMDMs (Figure S1B) nor impede TLR4 internalization triggered by LPS exposure (Figure S1C). Consequently, we conditioned BMDMs at pH 6.5 to further investigate the influence of acidic conditions on the inflammatory responses of macrophages.

The transcriptional response to LPS stimulation in macrophage is regulated by TLR4 signaling pathways, epigenetic mechanisms, and both feedback and feedforward genetic circuits.^20–23,48^ In response to LPS, transcriptionally induced genes can be categorized into primary response genes (PRGs) and secondary response genes (SRGs) depending on the reliance of the latter on newly synthesized proteins.^23^ PRGs can be further subdivided into early and late response groups based on activation kinetics. To evaluate the overall impact on the inflammatory response, we analyzed expression of early PRGs (*Cxcl1*, *Il1b, Nfkbia*, *Tnf*, *Tnfsf9*), late PRGs (*Ifnb1*) and secondary response genes (*Ifit1*, *Il6*, *Il12b*, *Saa3*) at 4 hours post LPS exposure.^49^ We found that conditioning BMDMs at pH 6.5 revealed a gene-specific impact on inflammatory genes that did not correlate with the classification of PRGs and SRGs (Figure 1D). The transcription of *Cxcl1, Ifit1, Nfkiba, Tnf* and *Vcam1* was relatively insensitive to pH, whereas *Edn1*, *Il1b*, *Il6*, *Il12b* and *Saa3* were significantly repressed at pH 6.5. Remarkably, the induction of *Ifnb1*, *Tnfsf9*, *Adm* and *Il23a* was greatly enhanced, showing an average of 20-fold increase at pH 6.5. Varying experimental conditions, including concentrations of LPS (10 to 1000 ng/mL) and duration of pH conditioning (4–12 hours) demonstrated a similar pattern of gene-specific regulation by acidic pH (Figure S1D, E). Moreover, time-course analyses of gene expression displayed a switch-like pattern among pH-regulated genes (Figure 1E). Notably, *Edn1* and *Adm*, which encode endothelin-1 and adrenomedullin respectively, exhibit differential activation at pH 7.4 and 6.5. These two peptides have antagonistic effect on blood vessels through vasoconstriction and vasodilation, respectively.^50^ The gene-specific sensitivity to the acidic environment and the regulation of opposing physiological functions suggest that macrophages may orchestrate a qualitatively different type of inflammatory response based on environmental cues, rather than merely adjusting the magnitude of immune activation.

### Known pH sensors cannot account for the pH-dependent inflammatory response

Among the known pH sensors, *Gpr65*, *Gpr68*, *Hif1a*, *Hif2a* are abundantly expressed in BMDMs and tissue resident macrophages (Figure 1F, S1F).^51^ We investigated pH-dependent inflammatory responses in BMDMs differentiated from *Gpr65*^−/−^, *Gpr68*^−/−^, *Hif1a*^*flox/flox*^ *lyz2*^*Cre*^ and *Hif2a*^*flox/flox*^ *lyz2*^*Cre*^ mice *in vitro*. However, our results indicated that neither *Gpr65*, *Gpr68*, *Hif1a* nor *Hif2a* alone was essential for the observed gene-specific, pH-dependent regulation (Figure 1G–I). At pH 6.5, both GPR65 and GPR68 activate the production of cyclic AMP (cAMP).^52^ To exclude the possibility of receptor redundancy, we treated BMDMs with 100 μM dibutyryl-cAMP, a cell-permeable analog of cAMP, and observe no significant effect on pH-dependent gene expression (Figure S1G). These findings suggested that the observed response was independent of established pH sensing mechanisms. Although acidic pH may broadly affect biochemical reactions and protein interactions, the specific activation and repression of genes at pH 6.5 imply that a general interference with transcription was unlikely. To further test this, we treated BMDMs with camptothecin, a DNA topoisomerase inhibitor that broadly represses the inflammatory response of macrophages by inhibiting Pol II.^53^ Camptothecin inhibited the activation for pH-insensitive and pH-sensitive genes (Fig S1H). Thus, we hypothesized that a novel specific pH-sensing mechanism controls inflammatory gene expression.

### A deconvolution model reveals pH-dependent inflammatory programs

**Figure 2.**
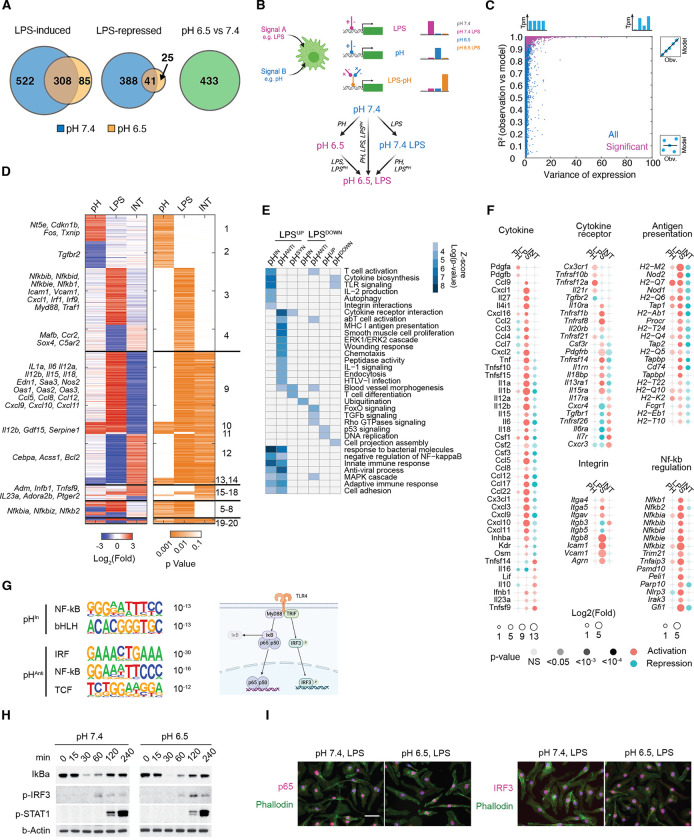
Deconvolution analysis revealed pH-dependent combinatorial control of inflammatory response. (**A**) Van diagrams of significantly regulated genes by LPS or acidic pH. Fc > 3 and q < 0.05. (**B**) Illustration of the linear deconvolution model to identify gene regulatory logics between LPS and acidic pH. (**C**) Evaluation of linear model fitting using expression variance and R-square for each gene. Blue marks all genes and magenta marks differentially expressed genes (FC > 3, q<0.05) in any pair of conditions. (**D**) Heatmap of inflammatory genes regulated by either LPS stimulation or acidic pH. Left, log2(fold) of model-inferred regulation by acidic pH alone (pH), LPS stimulation alone (LPS) and the interactions between acidic pH and LPS stimulation (LPS^PH^). Right, heatmap of p-values of each expression component, determined with a null hypothesis that the regulatory effect is less than 1.5-folds. Cluster groups (1–20) were determined based on expression component p-values. (**E**) Functional enrichment for LPS-induced or LPS-repressed genes that are pH-insensitive, pH-antagonistic or pH-synergistic. (**F**) Expression components of inflammatory cytokines, cytokine receptors, antigen presentation genes, integrins and regulators of NF-κB signaling. (**G**) Motif enrichment of LPS-induced genes that are pH-insensitive and pH-antagonistic. (**H**) Western blot of NF-κB, IRF3 and STAT1 signaling activation in BMDMs after LPS stimulation. (**I**) Immunofluorescence staining of p65 (pink) and IRF3 (pink) in BMDMs after LPS stimulation, 1 hour for p65 and 2 hours for IRF3.

We compared bulk RNA-seq of BMDMs at pH 7.4, pH 7.4 LPS 4 h, pH 6.5, and pH 6.5 LPS 4 h (Figure 2A) to comprehensively characterize the pH-dependent and pH-independent inflammatory response. Applying a stringent threshold (Fold change > 3 and q-value < 0.05), we identified 522 genes uniquely induced at pH 7.4 and 85 genes uniquely induced at pH 6.5, underscoring the gene-specific inflammatory response by acidic pH. However, among 308 genes induced commonly at both pH 7.4 and pH 6.5, many differed in their activation and expression quantitatively (Figure S2A). To gain a quantitative understanding of how the inflammatory response is regulated by acidic pH, we employed a linear deconvolution model originally developed to dissect regulatory interactions among transcription factors.^54,55^ This model conceptualizes that two signals, such as LPS stimulation and acidic pH, can control gene expression through three possible logics: LPS regulation independent of pH (LPS), pH regulation independent of LPS (pH), and the regulation dependent on the synergistic or antagonistic interactions between LPS and pH (INT) (Figure 2B). The sum of these 3 regulations (referred to as “expression components”) would equal the observed differential gene expression. Applying the linear deconvolution model allows integrating all four experimental conditions simultaneously to assess how LPS, pH, and their interactions contribute to the expression of each gene (Figure S2B). An expression component close to 0 indicates a lack of regulation, while a positive or a negative value indicates activation or repression, or that LPS and acidic pH act synergistically or antagonistically.

Overall, more than 92% of the genes significantly regulated by pH or LPS (Figure S2C) were well captured using the 3-component linear model (R^2^ > 0.9) (Figure 2C). We identified 1620 genes with at least one significant expression component (p<0.05, null hypothesis component > 2-fold), grouped in 20 clusters containing at least 5 genes each (Figure 2D, S2D). Among genes regulated by LPS and pH independently, Cluster 1–4 represent independent activation or repression by either acidic pH or LPS, while Clusters 5–8 represent combinations of these independent regulations. For example, Cluster 3 includes *Nfkbib*, *Nfkbid*, *Nfkbie* and Cluster 5 includes *Nfkbia* and *Nkfbiz*. They are induced by LPS independent of pH and have differences in pH-dependent basal expression (Figure S2F), all belonging to the NF-kb signaling pathway. Cluster 9–14 include genes regulated antagonistically by pH and LPS, including inflammatory cytokines (*Il6, Il12a, Il12b, Il18*), chemokines (*Ccl5, Ccl8, Ccl12, Cxcl9, Cxcl10, Cxcl11*), acute phase proteins (*Oas1, Oas2, Oas3, Saa3*) and inflammatory effectors (*Edn1, Nos2*). At last, group 15–18 include genes synergistically regulated by LPS and acidic pH, such as *Adm, Ifnb1, Tnfsf9, Il23a* and *Adora2b*. To examine potential immune functions enriched in pH-dependent and independent responses, we merged the 20 clusters into groups characterized as pH-insensitive, pH-antagonistic or pH-synergistic based on their interaction components (Figure S2D). Approximately 40% of LPS-regulated genes are pH-insensitive. Among pH-regulated genes, an antagonistic effect predominated in both LPS-induced and LPS-repressed response (88% and 86% respectively, Figure S2E). Since LPS induced transcriptional program serves as a model system for innate inflammatory response, we focus on the three groups of LPS induced genes: pH-insensitive (pH^IN^), pH-repressed (pH^ANTI^) and pH-synergistic genes (pH^SYN^). pH^IN^ genes were uniquely enriched in TLR signaling, T cell activation, integrin interactions, and showed strongest enrichment in innate immune response and antimicrobial defense (Figure 2E). The majority of LPS-induced integrins and NF-kb regulators are regulated by LPS independent of pH (Figure 2F). On the contrary, pH^ANTI^ genes were uniquely enriched in MHC I presentation, IL-1 signaling, chemotaxis, and showed the strongest enrichment in cytokine receptor interactions, antiviral and adaptive immune response (Fig. 2E). The activation of most antigen presentation genes and cytokines were strongly repressed by acidic pH (Figure 2F). Interestingly, pH^SYN^ genes, although fewest in number, were enriched in blood vessel morphogenesis and T cell differentiation (Figure 2E). These analyses revealed that the activation of innate defense programs is insensitive to pH, while the coordination and recruitment of other branches of the immune system are dependent on the tissue microenvironment.

TLR4-induced genes are regulated by two signaling adaptors, MyD88 and TRIF (Figure 2G).^48^ Myd88 recruits IL-1 receptor-associated kinases (IRAKs) and TRAF6, initiating the activation of the transcriptional activator NF-κB. Conversely, TRIF facilitates TRAF3-dependent activation of TBK1, which phosphorylates the transcription factor IRF3 to regulate interferon beta and other interferon-induced genes. We found that the consensus binding motif of p65 (NF-kB) was enriched in both pH^IN^ and pH^ANTI^ genes, whereas the IRF3 motif was only enriched in pH^ANTI^ genes (Figure 2G). However, LPS-induced degradation of the NF-κB inhibitory regulator Iκba, IRF3 phosphorylation as well as autocrine type-I Interferon signaling via STAT1 phosphorylation, all displayed comparable kinetics between pH 7.4 and pH 6.5 in BMDMs (Fig.2H). Furthermore, the nuclear localization of p65 and IRF3 was found to be comparable at both pH levels (Figure 2I). These findings suggest that pH-sensitive and pH-insensitive genes are differentially regulated at the level of transcription, rather than signal transduction.

### pH regulates gene expression at the chromatin level

**Figure 3.**
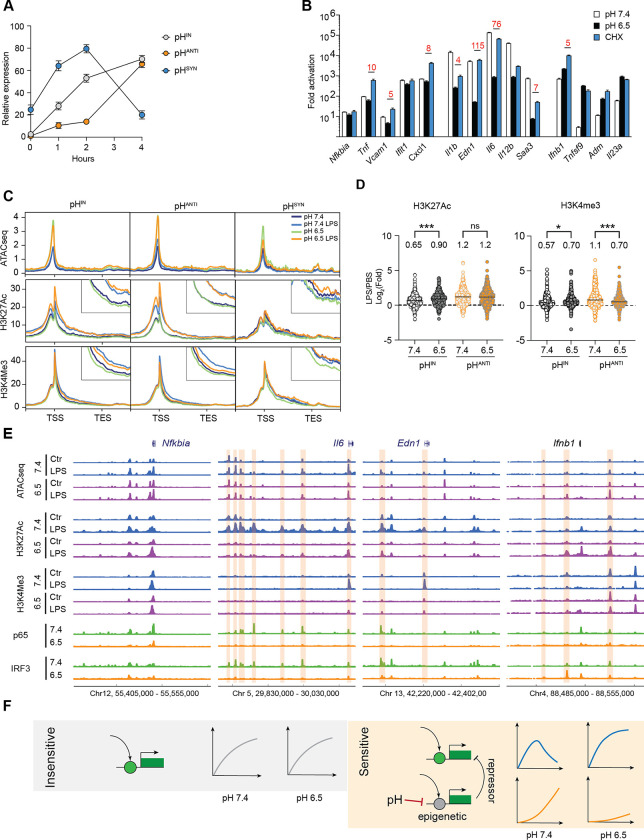
Transcription circuit and epigenetic control underlies environment-sensitive inflammatory response. (**A**) Expression kinetics of LPS-induced genes for top pH-insensitive (100), pH-antagonistic (100) and pH-synergistic groups (58). (**B**) Activation of inflammatory genes by LPS with 10 ng/mL for 4 hours, at pH 7.4, 6.5 or with 200 ng/mL cycloheximide (CHX). Fold change is calculated as LPS/untreated in each treatment condition. (**C**) Average profile of ATAC-seq and ChIP-seq of H3K27Ac and H3K4me3 of pH-regulated genes. Intersection image shows an enlarged profile around TSS. (**D**) Fold change of H3K27Ac and H3K4me3 ChIP-seq counts within 1kb around TSS for pH-insensitive and pH-antagonistic groups. Welch t-test and Holm-Sidak’s multiple comparisons test, ns p>0.05, * p<0.05, ***p<0.001. (**E**) Profile of ATAC-seq, H3K27 acetylation, H3K4 tri-methylation, p65 and IRF3 ChIP-seq at selected LPS induced genes. Orange boxes highlight regions with strong differences between pH 7.4 and pH 6.5. (**F**) model illustration of the activation of pH-sensitive and pH-insensitive inflammatory response.

IRF3 exhibited a delayed activation kinetics compared to NF-κB (Figure 2H). The observed enrichment of IRF3 motifs indicated possible difference in activation kinetics between pH^IN^ and pH^ANTI^ genes. At pH 7.4, we found that pH^IN^ genes reached full activation within 2 hours after LPS stimulation, whereas pH^ANTI^ genes showed minimal induction, only peaking at 4 hours (Figure 3A). Interestingly, pH^SYN^ genes demonstrated the most rapid and transient activation (Figure 3A, Figure S3A). The LPS-repressed genes also displayed a similar pH-dependent kinetics (Figure S3B). Therefore, acidic pH may act on a time-limiting step required for the activation of the pH^ANTI^ genes. LPS-induced SRGs typically have delayed activation due to the requirement for new protein synthesis,^49^ and are found to have a strong pH-dependence compared to PRGs in our data (Figure S3C). However, blocking protein synthesis in BMDMs with cycloheximide (CHX) did not affect pH^ANTI^ genes to the same extent as acidic pH (Figure 3B, S3D). On the other hand, the activation of pH^SYN^ genes was almost completely replicated by blocking protein synthesis (Figure 3B, S3D), indicating that an induced transcriptional repressor may be suppressed by acidic pH, thereby facilitating the elevated activation of pH^SYN^ genes.

A second possibility of a delayed activation kinetics involves chromatin remodeling. Given that acidic pH has been linked to modulation of histone acetylation and metabolism in cell lines,^56,57^ we performed ATAC-seq and ChIP-seq to examine chromatin accessibility and histone modifications (H3K27Ac and H3K4me3) associated with transcriptional activation. We first identified ATAC-seq peaks that were significantly induced by LPS (> 4-fold, pH 7.4 LPS vs pH 7.4) or homeostatically maintained (< 2-fold) at pH 7.4 across two biological replicates. At the induced peaks, we observed a comparable increase in ATAC-seq, H3K27ac and H3K4me3 induced by LPS at pH 7.4 and 6.5, while the homeostatic peaks remain unchanged among all examined conditions (Figure S3E). Intriguingly, ATAC-seq signals were mildly elevated at pH 6.5 (Figure S3E), suggesting that acidic pH does not restrict chromatin accessibility. The consistent levels of global H3K27Ac and K3K4me3 signals were also aligned with gene-specific control by acidic pH. Next, we analyzed chromatin changes specific to pH^IN^, pH^ANTI^ and pH^SYN^ genes (Figure 3C). Similar to the genomic profile, ATAC-seq signals at TSS were elevated at pH 6.5 across all three gene groups and demonstrated similar increase after LPS at pH 7.4 and 6.5. We observed no consistent difference in H3K27Ac, but LPS-induced increase of H3K4me3 was selectively reduced in pH^ANTI^ genes at pH 6.5, particularly within the gene body immediately downstream of TSS (Figure 3C,D), consistent with reduced transcriptional activity. Intriguingly, the pH^ANTI^ genes exhibited lower H3K27Ac and H3K4me3 signals at baseline, albeit a similar level of baseline expression and chromatin accessibility (Figure S3F). Overall, both the lack of pH-dependent difference in global histone modifications and the comparable regulation of chromatin accessibility and H3K27Ac changes at TSS suggest that acidic pH may impact the activity of distal regulatory elements to control gene activation.

The association between specific enhancers and genes is challenging to define at genome-wide in macrophages. We thus turned to investigate examples of pH-dependent and independent genes. At *Il6* and *Edn1* loci, gene induction was correlated with the activation of multiple distal enhancers spanning hundreds of thousands of base pairs—marked by increased ATAC-seq and broadened H3K27Ac peaks at pH 7.4 following 4 hours of LPS stimulation (Figure 3E). Notably, the H3K27Ac signals at these enhancers were completely abolished at pH 6.5, along with a reduction in chromatin accessibility and binding of p65 and IRF3 by ChIP-seq. In contrast to the examples of pH^ANTI^ genes, the pH^IN^ gene *Nfkbia* displayed consistent epigenetic marks across pH conditions, and the pH^SYN^ gene *Ifnb1* exhibited elevated H3K27Ac and H3K4me3 at gene TSS, as well as enhanced IRF3 binding at a −15kb enhancer (Figure 3E), arguing against the possibility that acidic pH blocks histone modification or epigenetic remodeling globally. To probe this further, we inhibited histone acetyltransferase (HAT) p300 with C646 and histone deacetylases (HDACs) with pan-inhibitor TSA.^58,59^ Overall, HAT inhibition resulted in a less profound effect than HDAC inhibition, yet both differed from the influence of acidic pH (Figure S3G). TSA strongly inhibited both pH^IN^ and pH^SYN^ genes (Figure S3D, S3G), and the activation of pH^ANTI^ genes required both HAT and HDAC activity (Figure S3G). Thus, combining epigenetic profiling and pharmacological perturbations, our data suggest a pH-sensitive epigenetic mechanism that specifically impacts the activation of inflammatory genes dependent on enhancer activation. Therefore, genes induced immediately by TLR signaling are insensitive to pH difference, while genes dependent on the activation of NF-κB, IRF3 and distal enhancers are regulated by the tissue microenvironment. A putative transcriptionally induced repressor belonging to the pH^ANTI^ group may be necessary to deactivate *Ifnb1*, *Adm* and other pH^SYN^ genes at pH 7.4; its absence at pH 6.5 leads to their prolonged and heightened activation (Figure 3F).

### Transcriptional condensates of BRD4 are sensitive to acidic pH

**Figure 4.**
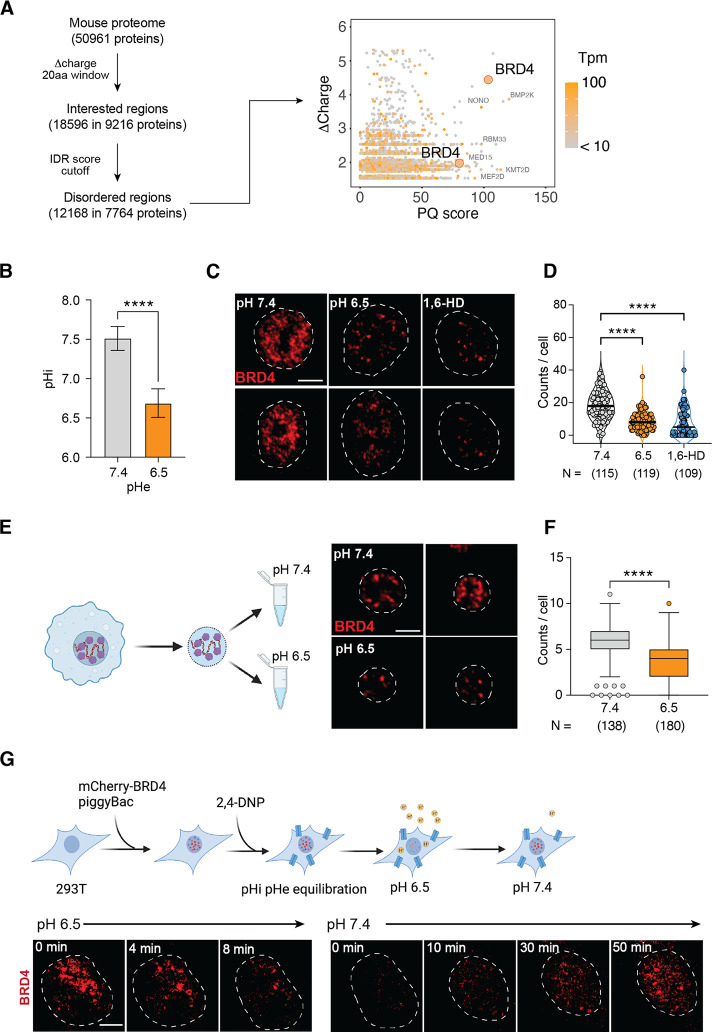
Acidic pH regulates BRD4 transcriptional condensates. (**A**) Diagram and results of the bioinformatic analysis of pH-sensitive peptide sequences in the mouse proteome. The 2D-plot shows side chain Δcharge and enrichment of proline and glutamine residues. Orange color indicates gene expression in BMDMs. (**B**) pHi measurement in BMDMs using pH-sensitive fluorescent probe SNARF-4F. **** p<0.0001, unpaired Student t test. (**C-D**) Immunofluorescent imaging (C) and quantification (D) of BRD4 in BMDMs cultured at pH 7.4, 6.5 for 4 hours, or treated with 10% 1,6-Hexanediol for 2 min. **** p<0.0001, One way ANOVA test. (**E-F**) Immunofluorescent imaging (E) and quantification (F) of BRD4 in isolated BMDM nuclei, conditioned in pH 7.4 and pH 6.5 buffer. **** p<0.0001, unpaired Student’s t test. (**G**) Time lapse imaging of BRD4 in live BMDMs in response to pH 6.5. N represents the number of cells analyzed for each condition in (E, G). Scale bar represents 2 μm.

Recent work has shown that the budding yeast SNF5, a core component of SWI/SNFchromatin remodeling complexes, was sensitive to intracellular pH (pHi).^60^ Two histidine residues on a disordered loop of *S. cerevisiae* SNF5 become protonated at pH 6.5 and this protonation disrupt the electrostatic interactions with nucleosomes and transcription factors.^60^ However, this disordered loop is not conserved in mammalian SNF5. Guided by its biochemical properties, we performed a bioinformatic screening to identify mammalian proteins that could mediate pH-dependent transcriptional response. Our screening was based on three assumptions: 1) a pH-sensitive protein must carry a significant difference in protonation between pH 7.4 and 6.5 (Δcharge), 2) any peptide region with significant Δcharge should be located near non-polar residues or disordered regions, with minimal prior structural constraints, and 3) the peptide region is enriched in prolines (P) and glutamines (Q), similar to the disordered loop in yeast SNF5. We first scanned 50,961 annotated protein sequences in the mouse genome (UniProt) to identify protein regions of interest (> 1 histidine residues in a stretch of 20 amino acids). We then filtered them based on IDR consensus score,^61^ and focused on Δcharge, PQ-enrichment and expression in BMDMs (Figure 4A). BRD4, a bromodomain-containing protein known for recognizing acetylated histones and regulating essential transcriptional programs in development and inflammation,^46^ was identified as a top candidate. It has two regions enriched for histidines, prolines, and glutamines (HPQ) (Figure 4B): AA 721–800, featuring a stretch of six consecutive histidines adjacent to a 40-residue non-polar region with 90% poly-PQ, and AA 1001–1080, containing nine histidines and 39 PQs. These regions have minimal net charge (Figure S4A) and are located within the C-terminal BRD4-IDR. The IDR-containing full length BRD4 is the most abundant isoform expressed in BMDMs (Figure S4B, C).

Using a pH-sensitive fluorescent dye SNARF,^62^ we found that the intracellular pH of BMDMs decreased to pH 6.6 in pH 6.5 medium (Figure 4B, S4D), exposing BRD4 to an acidic intracellular environment. Since BRD4 forms transcriptional condensates via its hydrophobic IDR,^35^ we hypothesized that pH-dependent protonation of histidine residues may disrupt the hydrophobic interactions of BRD4-IDR that are critical for condensate formation. At pH 7.4, endogenous BRD4 forms small and distinct foci in BMDMs, and these foci were substantially reduced at pH 6.5 (Figure 4C, D). Treatment with 10% 1,6-hexanediol, known to disrupt transcriptional condensates, reduced BRD4 foci similarly to acidic pH. Both BRD4 mRNA and protein expression remained stable at pH 6.5 (Figure S4E, F), demonstrating that condensates rather than BRD4 expression are regulated by pH. To discern potential roles of signaling and cytoplasmic contents, we isolated BMDM nuclei after gently lysing the plasma membrane and incubated them at pH 7.4 or 6.5 for 0.5 hours, followed by fixation and imaging (Figure S4G). We observed distinct BRD4 foci, although fewer in quantities in isolated nuclei than in living cells (Figure 4F). Despite the morphological difference, we found that BRD4 foci significantly decreased in acidic pH (Figure 4G), demonstrating nuclear intrinsic roles in regulating pH-dependent BRD4 condensates.

To further investigate the dynamics of BRD4 condensates, we generated a stable 293T cell line expressing a murine mCherry-BRD4 fusion protein (293T^BRD4^) for live-cell imaging. Initially, we noticed a heterogeneous response in mCherry-BRD4, likely due to variability in pH buffering capacity between cells. Treating 293T cells with a proton ionophore 2–4-Dinitrophenol (2,4-DNP) equilibrated extracellular and intracellular pH,^63^ significantly reducing the heterogeneity in BRD4 condensates (Figure S4H). To monitor the dynamic changes, we treated 293T^BRD4^ cells with 2,4-DNP for 30 min at pH 7.4 and abruptly switched the medium to pH 6.5. Remarkably, BRD4 condensates were mostly dissolved within 10–12 minutes, and upon returning to pH 7.4, BRD4 condensates reappeared and recovered in 1 hour (Figure 4H, S4I). In contrast, cells expressing only mCherry showed constant fluorescent signals unaffected by the pH changes (Figure S4J), consistent with previous studies on pH sensitivity of fluorescent proteins.^64^ Collectively, these findings demonstrated that BRD4 condensates are dynamically regulated in a pH-dependent manner within live cells, highlighting their potential role in regulating cellular responses to environmental shifts.

### Interference with BRD4 functions largely recapitulates pH-dependent responses

**Figure 5.**
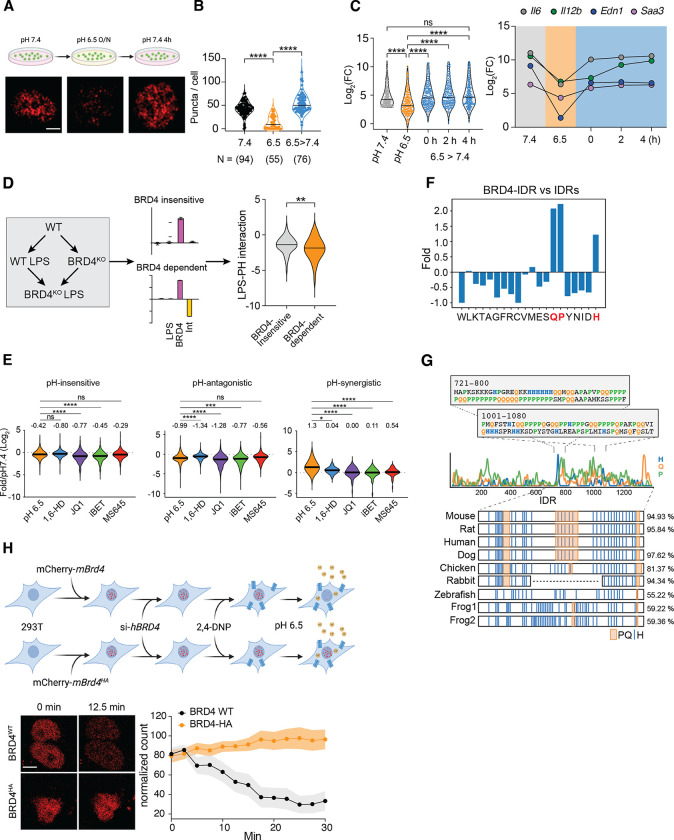
BRD4 is a pH sensor and regulates pH-dependent inflammatory response. (**A-B**) Immunofluorescent imaging (A) and quantification (B) of BRD4 in BMDMs. Brown-Forsythe and Welch ANOVA tests. (**C**) Violin plots of fold activation for pH-sensitive genes after restoring media pH from 6.5 to 7.4. The top 200 pH-repressed genes. Median of fold activation is labeled on violin plots. Kruskal-Wallis test with multiple comparisons. Only significant pairs are labeled. (**D**) RNA-seq deconvolution analysis of BRD4 KO BMDMs stimulated with 10 ng/mL LPS for 4 hours. LPS-induced BRD4-dependent and independent genes were analyzed for their pH-dependence. Unpaired Student’s t test. (**E**) Fold difference of pH-insensitive, -antagonistic or synergistic genes, at pH 6.5, with 1% 1,6-hexanediol or various BRD4 inhibitors. (**F)** Amino acid composition in the BRD4-IDR relative to that of all annotated IDRs in the mouse proteome. (**G**) Conservation of HPQ regions along the coding sequence of BRD4. Panels display amino acid sequences of the two HPQ regions within BRD4-IDR identified through bioinformatic screening. The distribution of H, P, Q residues, along with HPQ patterns in BRD4-IDR across different vertebrates are illustrated with sequence conservation. (**G**) Live-cell imaging of mCherry-BRD4^WT^ or mCherry-BRD4^HA^ 293T cells in response to pH 6.5. N represents the number of cells analyzed for each condition (B, C, D, E). ns, p>0.05, *p<0.05, **p<0.01, ***p<0.001, **** p<0.0001.

Given that pH-dependent changes in BRD4 condensates are reversible, we investigated whether the impact on inflammatory responses can be reversed after normalizing pH. After overnight incubation at pH 6.5, BRD4 condensates were significantly reduced, and were able to be fully restored after 4 hours at pH 7.4 (Fig. 5A, B). Alongside the changes in BRD4 condensates, pH-repressed genes regained activation nearly completely after re-conditioning at pH 7.4 (Fig. 5C). To test whether pH-dependent genes are regulated by BRD4, we analyzed the transcriptional response to LPS in *Brd4*^−/−^ BMDMs.^65^ We applied the deconvolution model to identify LPS-induced genes that are BRD4-dependent and independent, and found that the BRD4-dependent genes were more repressed under acidic conditions (Figure 5D). Similarly, competitive inhibitors targeting the bromodomains of BRD4 (JQ-1, iBET, and MS-645), or disrupting condensates with 1% 1,6-HD, consistently repressed pH^ANTI^ genes (Figure 5E). Thus, the pH-dependent inflammatory regulation is functionally linked to BRD4 condensates.

Histidines are responsible for the majority of Δcharge between pH 7.4 and 6.5. We found that BRD4-IDR is uniquely enriched for histidines (Figure 5F, S5A), as both full length BRD4 (Figure S5B) and IDRs from mouse proteome (Figure S5C) lack histidine enrichment. Moreover, the two HPQ regions identified from our bioinformatic screening are conserved among vertebrates (Figure 5G). To investigate whether protonation at histidine residues directly contribute to pH-dependent condensates, we synthesized a BRD4^HA^ mutant that swaps 21 histidines within BRD4-IDR to alanines, to eliminate most of pH-dependent Δcharge (Figure S5D). We generated stable 293T cell lines expressing comparable levels of mouse mCherry-BRD4^WT^ or mCherry-BRD4^HA^ (Figure S5D), and both murine BRD4 variants appeared in condensates in human 293T cells due to high sequence homology. To minimize the impact of endogenous human BRD4, we performed live-cell imaging at 24 hours after siRNA knock-down of hBRD4. In these experiments, we observed that the number of mBRD4^WT^ condensates (36 cells) began to decrease between 2–5 minutes at pH 6.5, stabilizing at ~25% of their original count by 30 mins. In contrast, replacing histidines with alanine in mBRD4^HA^ completely abolished the pH-dependent changes (45 cells) (Figure 5H). These data strongly support that BRD4 directly senses intracellular pH via its histidine-enriched IDR, thus dynamically regulating transcriptional condensates to control gene expression.

### Gene-specific mechanisms underlie pH-dependent regulation

BRD4 binds to and regulates both pH-sensitive and pH-insensitive genes (Figure 5E). Then how could pH-dependent BRD4 condensates specifically influence a subset of inflammatory genes? BRD4 recognizes histone acetylation via its bromodomains and recruits mediator complexes to active enhancers, facilitating the interaction between distal enhancers and promoters to activate transcriptional machinery.^41^ Given the distinct changes at enhancers of pH-sensitive genes, we hypothesize that pH-dependent condensates facilitate remodeling and activation of enhancers, and thus control gene activation that strongly depends on these processes.

**Figure 6.**
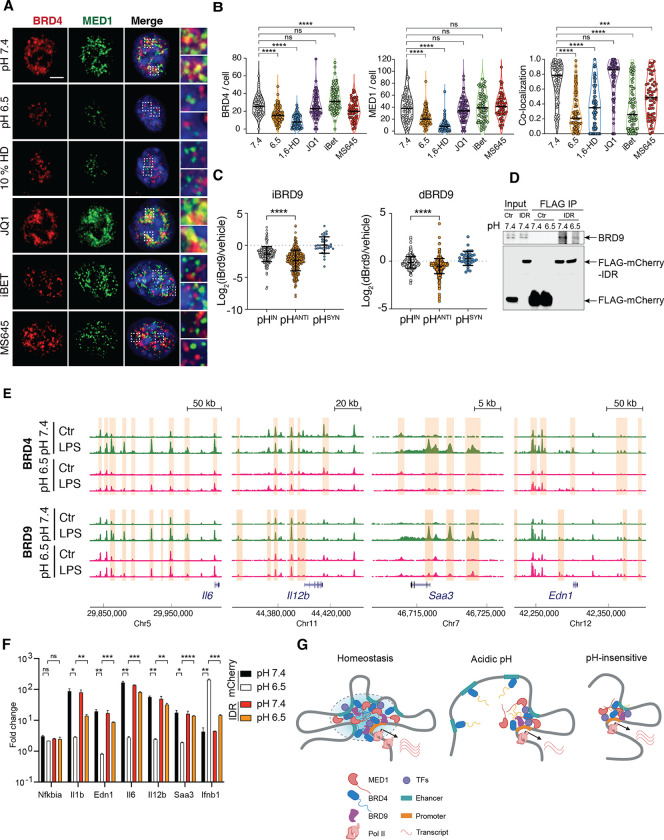
Gene-specific regulation is mediated by BRD4-MED1 transcription condensates and BRD9. (A-B) BRD4 and MED1 in BMDMs at pH 7.4, at 6.5, in the presence of 10 % 1,6-HD, JQ-1, iBET and MS645. (A) Immunofluorescent imaging (B) Quantification of puncta and co-localization. One way ANOVA. (**C**) Fold change of LPS induced gene expression in the presence of BRD9 specific inhibitor (iBRD9) of degrader (dBRD9). (**D**) Western blot analysis of Co-IP between BRD4-IDR and BRD9. FLAG-mCherry was fused with BRD4-IDR for IP with total cell lysate, with FLAG-mCherry as control (Ctrl). (**E**) ChIP-seq of BRD4 and BRD9 in BMDMs at pH-regulated genes. Orange boxes highlight regions with significant differences in BRD4 or BRD9 binding. (**F**) Fold change of pH-dependent inflammatory genes in iBMDMs overexpressing BRD4-IDR after 6 hours 100 ng/mL LPS at pH 7.4 or 6.5, normalized to unstimulated conditions respectively. Mean+/− standard deviation (STD). Unpaired t-test, Holm-Sidak’s test for multiple comparisons. (**G**) Model diagram of pH-dependent regulation of BRD4 condensates. In (B), (D), (F) ns p>0.05, *** p<0.001, **** p<0.0001.

First, bridging enhancer activation to gene promoters requires mediators.^66^ Both BRD4 and MED1 form liquid-liquid phase condensates, and co-localize at distinct nuclear foci to recruit RNA polymerase II.^35^ At pH 7.4, endogenous MED1 formed condensates in BMDMs and co-localized with BRD4 (Figure 6A). At pH 6.5, however, these condensates were substantially reduced, despite MED1 lacking a HPQ enriched IDR (Figure S6A, B). Notably, the remaining puncta of BRD4 and MED1 were partitioned spatially, contrasting with the strong colocalization seen at pH 7.4 (Figure 6A, B). These pH-dependent changes were also aligned with chemical disruption of condensates by 1,6-HD (Fig. 6A, B). Since reduction of H3K27Ac was observed at pH-sensitive genes, we thus tested whether interfering binding to histone acetylation results in similar perturbation of BRD4 condensates. Surprisingly, BRD4 and MED1 condensates were differentially affected by JQ1, iBET and MS645, although all of them inhibited LPS-induced inflammatory programs (Figure 5E). The contrasting phenotypes between acidic pH and BRD4 inhibitors also implied that altered chromatin recruitment was not the cause for pH-dependent dissolution of transcription condensates. This is corroborated by comparable levels of global histone acetylation (Figure S3E), binding of BRD4 at house-keeping genes (Figure S6C), and previous findings suggesting that the reliance on histone acetylation may vary by cell type.^35,43,67^ Thus, acidic pH disrupts the formation of transcriptional condensates containing both BRD4 and MED1, two essential components of enhancer-regulated transcriptional activation.

Second, enhancer activation often requires chromatin remodeling. Bromodomain-containing protein 9 (BRD9), a subunit of the noncanonical SWI/SNF chromatin remodeling complex (ncBAF), co-localizes with BRD4 at the enhancers and promoters of interferon-stimulated secondary response genes enriched with NF-κB and IRF3 binding motifs.^68^ The inhibition of BRD4 with JQ1 displaces BRD9 in both BMDMs and embryonic stem cells ^68,69^ without directly inhibiting BRD9 *in vitro*.^70^ These evidences suggest that BRD4 can recruit BRD9-containing ncBAF complex for enhancer activation. We hypothesized that disruption of BRD4 condensates at acidic pH leads to a defective recruitment of BRD9 to regulate pH-sensitive genes. Indeed, in the presence of a specific BRD9 inhibitor (BRD9i) or a BRD9 degrader (dBRD9), pH^ANTI^ genes exhibited a significantly reduced activation in comparison to pH^IN^ genes (Figure 6C).^68^ ChIP-seq analysis of BRD4 and BRD9 revealed a significant reduction in LPS-induced recruitment at the enhancers and promoters at pH 6.5, particularly for pH-sensitive genes such as *Il6, Il12b, Saa3, Edn1* (Figure 6E). Among BET family proteins, only BRD4 contains HPQ regions, suggesting that pH-dependent BRD9 recruitment is likely mediated through BRD4 (Fig. S6A, B). A recent proteomic analysis further suggested that the IDR of BRD4 may directly interact with BRD9.^71^ We thus expressed FLAG-IDR^BRD4^-mCherry to test whether the recruitment BRD9 is affected by pH. We observed that FLAG-IDR^BRD4^-mCherry were integrated into BRD4 condensates (Figure S6D). Using Co-immunoprecipitation (Co-IP), we found that the co-association of BRD9 to BRD4 was substantially reduced at pH 6.5 (Figure 6D). In connection with the differential regulation of distal enhancers of pH-sensitive genes (Figure 3E), these data suggest that pH-dependent BRD4 condensates may regulate a subset of inflammatory response in macrophages by directly recruiting the non-classic BAF complex to activate enhancer.

BRD4, MED1 and enhancer activation are crucial for RNA Pol II elongation. We speculate that Pol II elongation at pH-sensitive genes was selectively inhibited at acidic pH conditions, and activating elongation may mitigate the suppressive inflammatory response. The C-terminal fragment of BRD4 interacts with pTEFb and has been shown to release paused Pol II independent of the BET bromodomains.^72^ We thus tested the possibility of rescuing pH-dependent repression using BRD4-IDR. Leveraging the immortalized BMDM cell line (iBMDMs), we identify pH sensitive genes consistent between iBMDMs and BMDMs (Figure S6E). Over-expression of mCherry-IDR^BRD4^ significantly reversed the pH-dependent repression and reduced the synergistic induction of *Ifnb1*, while maintaining proper activation of pH-insensitive genes (Figure 6F). Thus, we concluded that transcription condensates integrate environmental signals to orchestrate a gene-specific regulation of inflammatory response facilitated by chromatin remodeling (Figure 6G). Leveraging BRD4-IDR can reverse environment-dependent repression of inflammatory responses.

### pH sensing by BRD4 mediates feedback control of inflammatory activation

**Figure 7.**
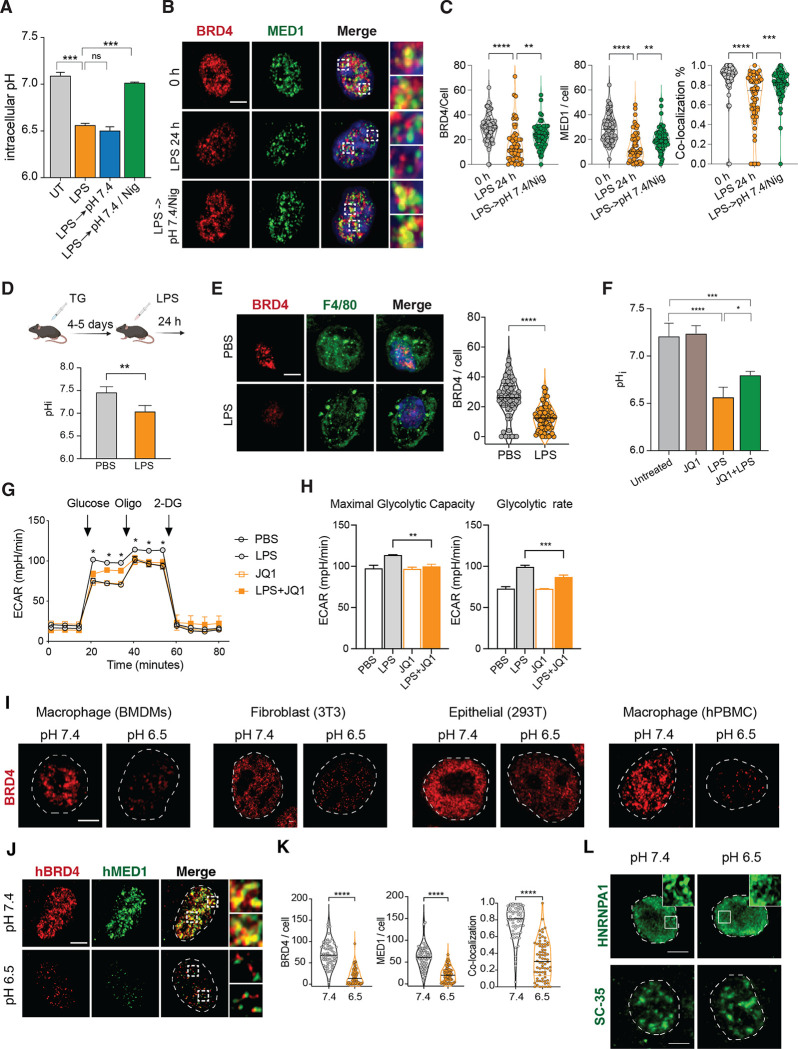
pHi sensing via BRD4 acts as negative feedback to modulate pH homeostasis and inflammatory response. (**A)** pHi in BMDMs after LPS stimulation in various indicated conditions, pairwise comparison with Kruskal-Wallis. (**B-C**) Immunofluorescent imaging (B) and quantification (C) of BRD4, MED1 and co-localization. one way ANOVA (F). (**D-E**) pHi and BRD4 measurement of thioglycollate-induced peritoneal macrophages *in vivo*, 24 h after i.p. injection with PBS or 3 mg/kg LPS. pH quantification, unpaired Student’s t test (D), Immunofluorescent imaging and quantification of F4/80+ macrophages, Mann-Whtiney test (E). (**F**) pHi of BMDMs treated with 100 ng/mL LPS for 24 h and/or 0.5 μM JQ1 for 8 h, one way ANOVA. (**G**) Seahorse analysis of glycolytic activity in BMDMs treated with 100 ng/mL LPS and/or 0.5 μM JQ1 for 8 h, one way ANOVA. (**H**) Glycolytic capacity and rate of BMDMs treated with 100 ng/mL LPS +/− 0.5 μM JQ1 for 8 h, one way ANOVA. (**I, L**) Immunofluorescent imaging of BRD4 (I), HNRNPA1 or SRSF2 (L) phase condensates in murine and human cells. (**J, K**) BRD4 and MED1 puncta in human macrophages at pH 7.4 and pH 6.5. (J) Immunofluorescent imaging, (K) Quantification of puncta and co-localization. Mann-Whtiney test. p>0.05, *p<0.05, **p<0.01, ***p<0.001, **** p<0.0001.

To understand the potential functions of pH-dependent transcriptional condensates during inflammation, we revisited the change of pH upon stimulation with LPS. Interestingly, we found that the pHi of BMDMs quickly decreased and plateaued after 8 hours (S7A), in addition to acidifying the extracellular environment (Figure 1B). Such acidic pHi cannot be simply restored by conditioning LPS-stimulated BMDMs in pH 7.4 medium up to 8 hours (Figure 7A), suggesting that an intrinsic program maintains the acidic intracellular environment. Consequently, BRD4 and MED1 condensates were significantly reduced simply by activating innate sensing pathways (Figure 7B and C). We found that subjecting LPS-treated BMDMs to a 5 min pulse of 500 nM nigericin and100 mM KCl followed by conditioning at pH 7.4 can largely restore the pHi to 7.0 (Figure 7A). Following this treatment, the increase in pHi restored both BRD4 and MED1 condensates as well as their colocalization (Figure 7B and C), demonstrating that low pHi induced by LPS *in vitro* is sufficient and necessary to inhibit transcriptional condensates. *In vivo*, we examined the response of thioglycolate-induced peritoneal macrophages (pMac) to i.p. Injection of LPS. Thioglycolate induces the expansion of CD11b^Int^ F4/80^low^ MHC-II^+^ pMac (Figure S7B).^73^ Although the number of pMac decreased after LPS treatment, the remaining pMac from peritoneal cavity at 24 hours displayed a significant reduction in pHi and BRD4 puncta after a moderate dose of LPS, compared to the PBS-treated controls (Figure 7D,E). Altogether, these data demonstrated that inflammatory activation of macrophages *in vitro* and *in vivo* adopts an acidic intracellular environment that is necessary and sufficient to regulate transcription condensates. Since acidic pH represses the inflammatory response in macrophages, our data suggest that sensing pH via BRD4-containing transcription condensates function as negative feedback to control the inflammatory response.

To explore the role of pH-sensing by BRD4 in cellular physiology, we reasoned that a pH sensor may regulate cellular processes to control pHi, similar to a homeostatic controller (Figure S7C).^1,3^ Thus, we tested whether BRD4 regulates LPS-induced intracellular acidification. Interestingly, JQ-1 treatment alleviated LPS-induced acidification in BMDMs by 0.23 pH unit (1.44-fold of protons) without impacting naive BMDMs (Figure 7F). Using seahorse assay, we observed that both glycolytic capacity and glycolytic rate in BMDMs were enhanced by LPS stimulation and this increase in glycolysis was impaired by JQ-1 (Figure 7G, H). Similarly, BMDMs activated at pH 6.5 also displayed reduced glycolytic functions (Figure S7D). Mechanistically, we found that hexokinases (HK1, HK2, HK3) that catalyze the phosphorylation from glucose to phopho-6-glucose, the first chemical reaction of converting glucose to energy, were transcriptionally induced by LPS in macrophages in a BRD4-dependent manner (Figure S7E). Thus, BRD4 acts as a sensor of pHi to modulate both transcriptional and metabolic inflammatory response.

### BRD4 is a generic pH sensor in multiple cell types

Finally, given that BRD4 is a crucial gene expressed in all cell types, we investigated if its pH-dependent regulation is a general phenomenon across various cell types and species. We observed that murine and human primary macrophages, as well as stromal and epithelial cell lines, all exhibit pH-dependent BRD4 condensates (Figure 7I). In particular, BRD4 and MED1 in human macrophages derived from peripheral blood mononuclear cells displayed strong pH dependence (Figure 7J, K). It is worth noting that different cell types may differ in their sensitivity to extracellular pH and likely exhibit cell-type dependent response under acidic environment *in vivo*. Moreover, we found that other types of nuclear condensates, such as RNA granules and stress granules were not sensitive to the pH conditions examined here (Figure 7L). Thus, pH-sensing by BRD4 condensates establishes a unique mechanism to integrate extracellular and intracellular environments in regulating inflammatory response and beyond.

## Discussion

During inflammation, extreme perturbations in cellular or tissue microenvironment signals strong deviation from tissue homeostasis. Adjusting immune responses to the changes in the microenvironment provides an adaptable strategy for the immune system to calibrate inflammatory responses to the consequences of inflammation.^2,3^ We found that environmental pH regulates switch-like transcriptional responses encoding distinct inflammatory programs in macrophages. Mechanistically, we discovered that the epigenetic regulator BRD4 acts as a novel intracellular pH sensor, regulating transcriptional condensates via enriched histidines within the intrinsically disordered region. pH-dependent and independent inflammatory responses differ in their activation kinetics, requirement for chromatin remodeling, and dependence on non-canonical SWI/SNF chromatin remodeling complex. Interestingly, innate activation of macrophage triggers and maintains an acidic intracellular environment, which is necessary and sufficient to disrupt BRD4 condensates *in vitro* and *in vivo*. We propose that BRD4-dependent transcriptional condensates provide a novel platform for cells to integrate environmental information in cellular decisions.

Hundreds of genes are regulated by the LPS-induced inflammatory response in macrophages.^22,23^ These genes are activated by transcription factors immediately downstream of TLR4 signaling and reinforced by feedforward autocrine signals and secondary transcription factors.^49,74^ This results in a robust, dynamic, and deterministic transcriptional response studied by numerous laboratories that is nearly “hardwired” to TLR4 activation under well-controlled cell culture conditions. However, the extensive pH-dependent transcriptional reprogramming and switch between alternatively induced genes suggests an alternative paradigm that the innate inflammatory response is adaptable and tunable. Interestingly, not all inflammatory genes respond to environmental changes to the same degree. We found that inflammatory genes involved in antimicrobial defense are induced immediately in response to LPS independent of pH (Figure 2D). The activation of these genes meets the basic demand for clearing microbial infections, and is likely programmed at their gene promoters to scale the response based on inflammatory inputs. Conversely, genes that may function to amplify the inflammatory cascade, such as coordinating the innate and adaptive immunity, bridging antiviral response and communicating with tissue stromal and parenchymal, are thought to carry a high cost and are found to be sensitive to the environment. Their activation is tightly controlled by complex regulatory mechanisms, including combinatorial transcription factors, chromatin remodeling and enhancer-promoter interactions as “tuning knobs” of inflammation.^75,76^ Dissecting pH-dependent inflammatory response in macrophages highlights a new way to understand the logic of integrating immunological and environmental signals in inflammation.

Histidine residues are well recognized for their pH-dependent ionization near physiological pH.^77,78^ A shift in pH from 7.4 to 6.5 is expected to cause a charge difference of 0.2, or 2 out of 10 histidine residues transitioning from neutral to positively charged on average. Known pH sensors, such as GPCRs and ASICs, are thought to contain specific histidine-containing domains to transduce extracellular pH signals.^79^ Distinct from these well-structured proteins, we described that the disordered and conserved BRD4-IDR acts as a physiological intracellular pH sensor. Notably, a motif of six consecutive histidine residues—commonly used as an affinity tag for protein purification— surrounded by prolines and glutamines, likely mediates pH-sensing capacity. This HPQ region can create an unstructured and hydrophobic local environment to amplify the biophysical impact of pH-dependent ionization on histidines. The multivalent interactions intrinsic to IDR sequences provide compounding effects that lead to an ultrasensitive phase transition in transcriptional condensates. The peptide feature of concentrated histidines embedded in a nonpolar and unstructured region may inspire identifying additional pH-sensitive proteins or engineering synthetic pH-dependent regulators. Indeed, recent research has shown that consecutive histidine residues can be used to engineer synthetic pH-sensitive peptides that respond to a pH change of just 0.3 units.^80^ Other endogenous proteins carrying pH-sensitive motifs may exhibit unique and cell-type-specific functions based on the tissue and cellular microenvironment.

Nuclear condensates have recently emerged as a unique and intriguing feature of many transcription and chromatin regulators.^26,28,29^ Recent studies just began to uncover the mechanisms that contribute to condensate formation, partitioning and specific roles in gene regulation. In MED1, the IDR features alternating blocks of positively and negatively charged residues, which recruit subunits of RNA Pol II and exclude negative transcription regulators.^30^ The chromatin remodeler cBAF subunit ARID1A/B contains blocks of alanine, glycine, and glutamine essential for interactions with a network of transcriptional regulators.^32^ These newly identified partitioning features suggest that transcriptional condensates are heterogenous and can be self-regulated by their internal components. However, whether cellular or environmental signals dictate transcriptional condensates remain unknown. Our work demonstrated for the first time that the pH environment controls the formation of transcriptional condensates in primary human and mouse cells. BRD4 acts as sensors for the pH, with its histidine-enriched IDR mediating pH-dependent condensate disruption and formation. Furthermore, we observed that condensate-dependent transcriptional responses only partially overlap with BRD4-dependent responses. In fact, many pH-insensitive and pH-synergistic genes are dependent on BRD4, highlighting a discrepancy between BRD4 condensates and BRD4 in activating gene expression. We observed that inflammatory genes relying on chromatin remodeling at distal enhancers presented the most dependence on pH and BRD4 condensates (e.g. *Il6*, *Il12b*, *Edn1*). These genes tend to have multiple enhancers or a long-stretch of enhancers induced after LPS, mimicking super enhancers found at the lineage-determining genes. These enhancers are likely responsible for the establishment of inducible chromatin loops with gene promoters (data not shown). We propose that BRD4-containing transcriptional condensates are crucial for remodeling and activating distal enhancers, thereby establishing new enhancer-promoter contacts that facilitate the release of paused Pol II for efficient transcriptional elongation. Genes requiring such mechanisms for activation are specifically tuned by pH. Although the understanding of gene-specific enhancer activation remains incomplete, the mitigation of pH-dependent repression by BRD4-IDR suggests that targeting pH-dependent condensate pathway may help reverse immune suppression associated with acidic microenvironment.

Last, since BRD4 is universally expressed, pH-sensitive mechanisms may be broadly applicable across both immune and non-immune cell types to mediate responses to inflammatory cues under acidic conditions. In immune cells, these mechanisms could determine the nature of the inflammatory response, as well as cell differentiation and polarization programs. In non-immune cells, they may selectively diminish the cells’ responsiveness to the same inflammatory cues, potentially providing a means for cells to tolerate high levels of inflammation that would otherwise be detrimental.

### Limitations

Significant additional investigation will be needed to define the similarities and differences between these frameworks and the specific inflammatory response triggered by other pattern recognition receptors, in other types of tissue resident macrophages naturally present in different tissues, or other types of primary cell types and immortalized cell lines. In particular, we observed variable sensitivity to perturbation of extracellular pH among different cell types. The condensate-dependent transcriptional control may be cell-type specific within a local tissue microenvironment. Moreover, it remains to be determined how BRD4 transcriptional condensates specifically regulate enhancer activity and/or loop interactions between enhancers and promoters, or how chromatin recruitment, histone modification and co-condensate molecules impact the sensitivity of pH-dependent regulation. Furthermore, it remains an active investigation whether genetic or synthetic approaches that reverse pH-dependent transcriptional condensate present a viable strategy to modulate immune activity in disease associated with local or systemic acidosis.

## Supplementary Material

Supplement 1

## Highlights

- Acidic pH regulates a switch-like gene-specific inflammatory response in macrophages
- Acidic pH impacts chromatin remodeling and transcription circuits to control inflammatory programs
- BRD4 transcriptional condensates are regulated by intracellular pH via pH-sensitive motifs located within the intrinsically disordered region
- Tissue inflammation decreases intracellular pH and disrupts BRD4 condensates as a negative feedback
