# Supplementary material for: Control of Inflammatory Response by Tissue Microenvironment
Source: bioRxiv. 2025 May 30:2024.05.10.592432. Originally published 2024 May 14. Preprint. [Version 2] doi: 10.1101/2024.05.10.592432 (PMC11118380; doi:10.1101/2024.05.10.592432)
Supplement: Supplement 1 [file NIHPP2024.05.10.592432v2-supplement-1.pdf]

bioRxiv preprint doi: https://doi.org/10.1101/2024.05.10.592432; this version posted May 30, 2025. The copyright holder for this preprint (which was not certified by peer review) is the author/funder, who has granted bioRxiv a license to display the preprint in perpetuity. It is made available under aCC-BY 4.0 International license.

Supplementary figures

Figure S1.

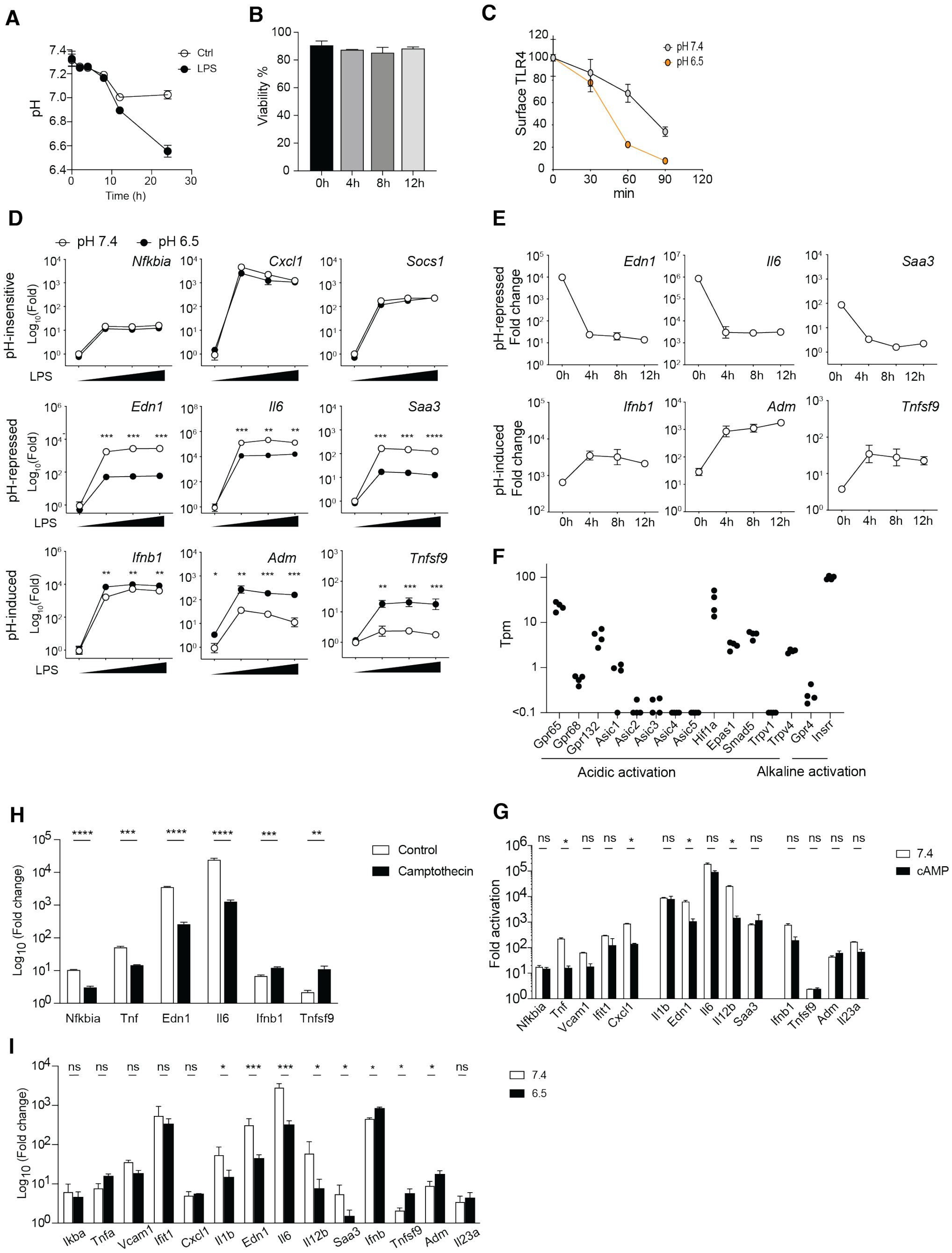

(**A**) Extracellular pH of BMDMs stimulated with 100 ng/mL LPS *in vitro*. Mean + STD. (**B**) Viability of BMDMs in acidic medium. Cell viability was assayed by flow cytometry using Annexin V and propidium iodide staining. Cells were gated for single cells based on FSC and SSC, and then gate for Annexin V$^-$and PI$^-$ for viable cells. (**C**) Internalization of TLR4 measured by flow cytometry. The median fluorescent intensity of each sample is subtracted of background (unstained control) and normalized to the surface TLR4 staining before LPS stimulation. Mean +/- STD. (**D**) Fold activation of inflammatory genes in BMDMs at 10-1000 ng/mL LPS treatment for 4 h. Unpaired t-test, Holm-Sidak's test for multiple comparisons. mean +/- STD. (**E**) Fold activation of inflammatory genes in BMDMs after conditioning under acidic pH for 0-12 h. (**F**) Expression of known pH sensors in BMDMs from bulk RNA-seq. (**G-I**) Fold activation of inflammatory response genes after 4 h 10 ng/mL LPS, in the presence of 100 μM cAMP (G) or 5 μM Camptothecin (H) in WT BMDMs, or at pH 7.4 or 6.5 in *Nlrp3*$^{-/-}$ BMDMs (I). Mean+/- STD. Unpaired Student's t test, Holm-Sidak's test for multiple comparisons. ns p>0.05, * p<0.05, ** p<0.01, *** p<0.001.

bioRxiv preprint doi: https://doi.org/10.1101/2024.05.10.592432; this version posted May 30, 2025. The copyright holder for this preprint (which was not certified by peer review) is the author/funder, who has granted bioRxiv a license to display the preprint in perpetuity. It is made available under aCC-BY 4.0 International license.

Figure S2.

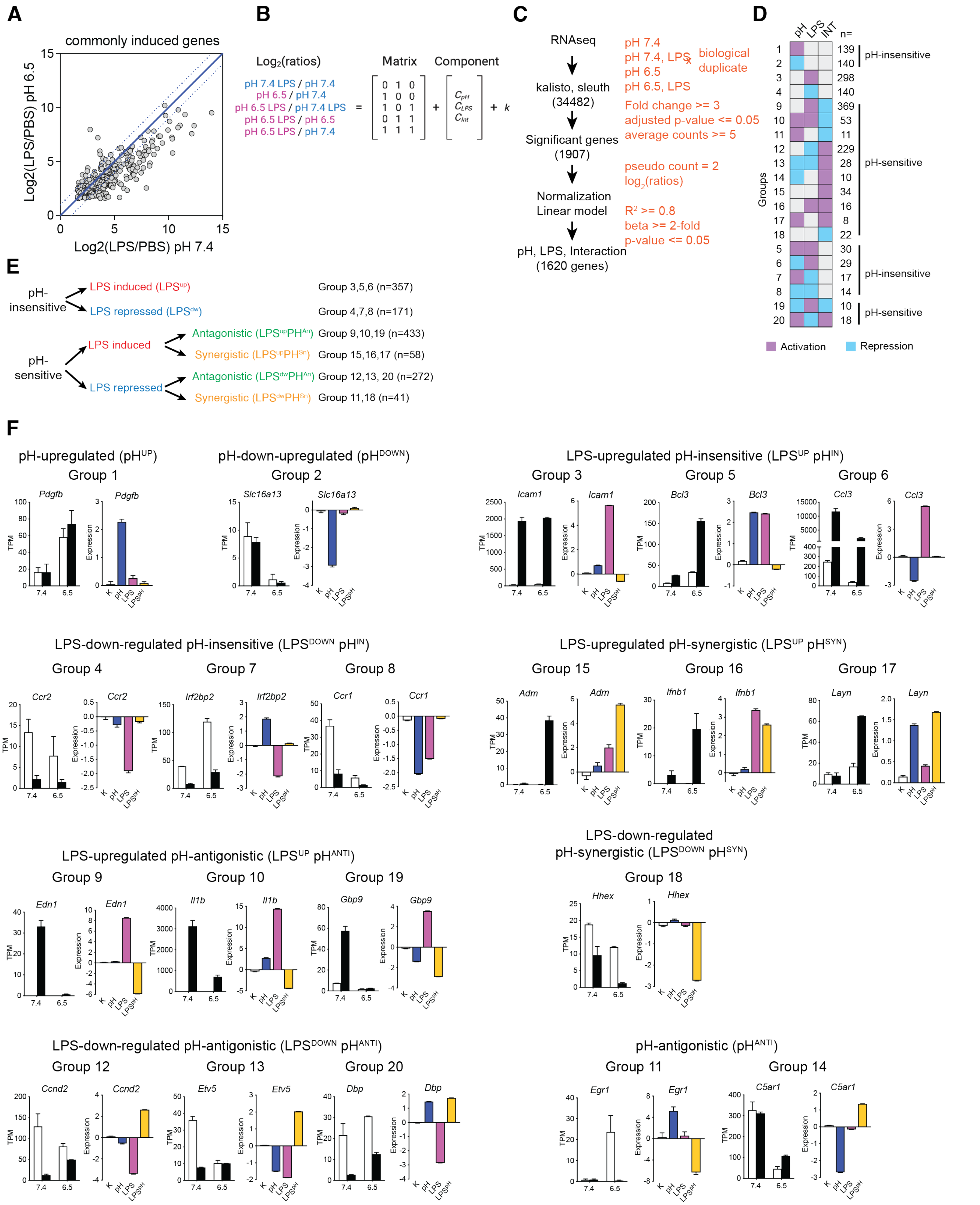

(**A**) Log2 (FC) of LPS-induced genes at both pH 7.4 and pH 6.5. Blue dot lines indicate the range of 2-fold variation. (**B**) Linear deconvolution matrix for dissecting the interactions between LPS and pH. (**C**) Analytic pipeline and thresholds applied to identify pH- and LPS-regulated genes. (**D**) Illustration of pH-sensitive and pH-insensitive genes in 20 identified clusters. (**E**) illustration of combining cluster groups into pH^{IN}, pH^{ANTI}, and pH^{SYN} groups. (**F**) Examples of each 20 gene clusters based on deconvoluted gene expression components.

bioRxiv preprint doi: https://doi.org/10.1101/2024.05.10.592432; this version posted May 30, 2025. The copyright holder for this preprint (which was not certified by peer review) is the author/funder, who has granted bioRxiv a license to display the preprint in perpetuity. It is made available under aCC-BY 4.0 International license.

Figure S3.

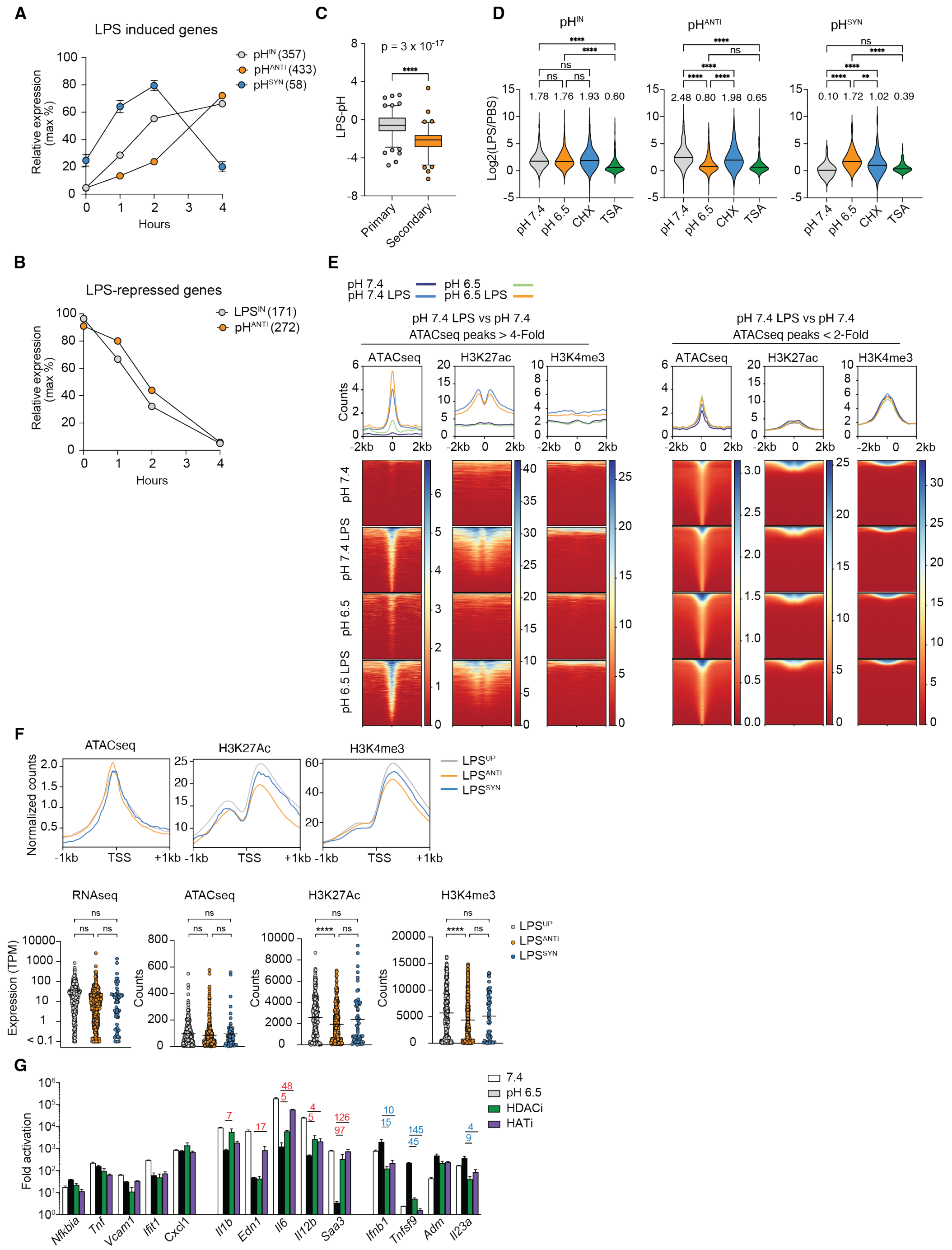

(**A**) Expression kinetics of all LPS-induced genes for pH-insensitive (357), pH-antagonistic (433) and pH-synergistic (58) groups. (**B**) Expression kinetics of all LPS-repressed genes for pH-insensitive (171) and pH-antagonistic (272) groups. (**C**) The LPS-PH component of primary and secondary response genes in response to LPS in BMDMs identified in Tong et al., 2016. (**D**) Violin plots of the fold change of gene expression between 10 ng/mL LPS at 4 hours and PBS control for the indicated conditions. CHX, 200 ng/mL cycloheximide; TSA, 50 mM TSA. The numbers above violin plots indicate the group median. Friedman ANOVA, Dunn's test for multiple comparisons. (**E**) Genomic profile of ATAC-seq, ChIP-seq of H3K27Ac and H3K4me3 marks at pH 7.4, pH 7.4 LPS 4 h, pH 6.5, pH 6.5 LPS 4 h, for ATAC-seq peaks with significant increase after LPS at pH 7.4 (left), or with less than 2-fold change at pH 7.4 (right). (**F**) Average profile of ATAC-seq, ChIP-seq of H3K27Ac and H3K4me3 marks AT pH 7.4 for LPS^{IN}, LPS^{ANTI} and LPS^{SYN} groups (top) and comparison of RNA-seq, ATAC-seq, H3K27Ac and H3K4me3 signals for for LPS^{IN}, LPS^{ANTI} and LPS^{SYN} groups (bottom). (**G**) Activation of inflammatory genes by 10 ng/mL LPS for 4 h, with 50 mM TSA (HDAC inhibitor) or 10 μM C646 (HAT inhibitor). ns p>0.05, ** p<0.01, **** p<0.0001.

bioRxiv preprint doi: https://doi.org/10.1101/2024.05.10.592432; this version posted May 30, 2025. The copyright holder for this preprint (which was not certified by peer review) is the author/funder, who has granted bioRxiv a license to display the preprint in perpetuity. It is made available under aCC-BY 4.0 International license.

Figure S4.

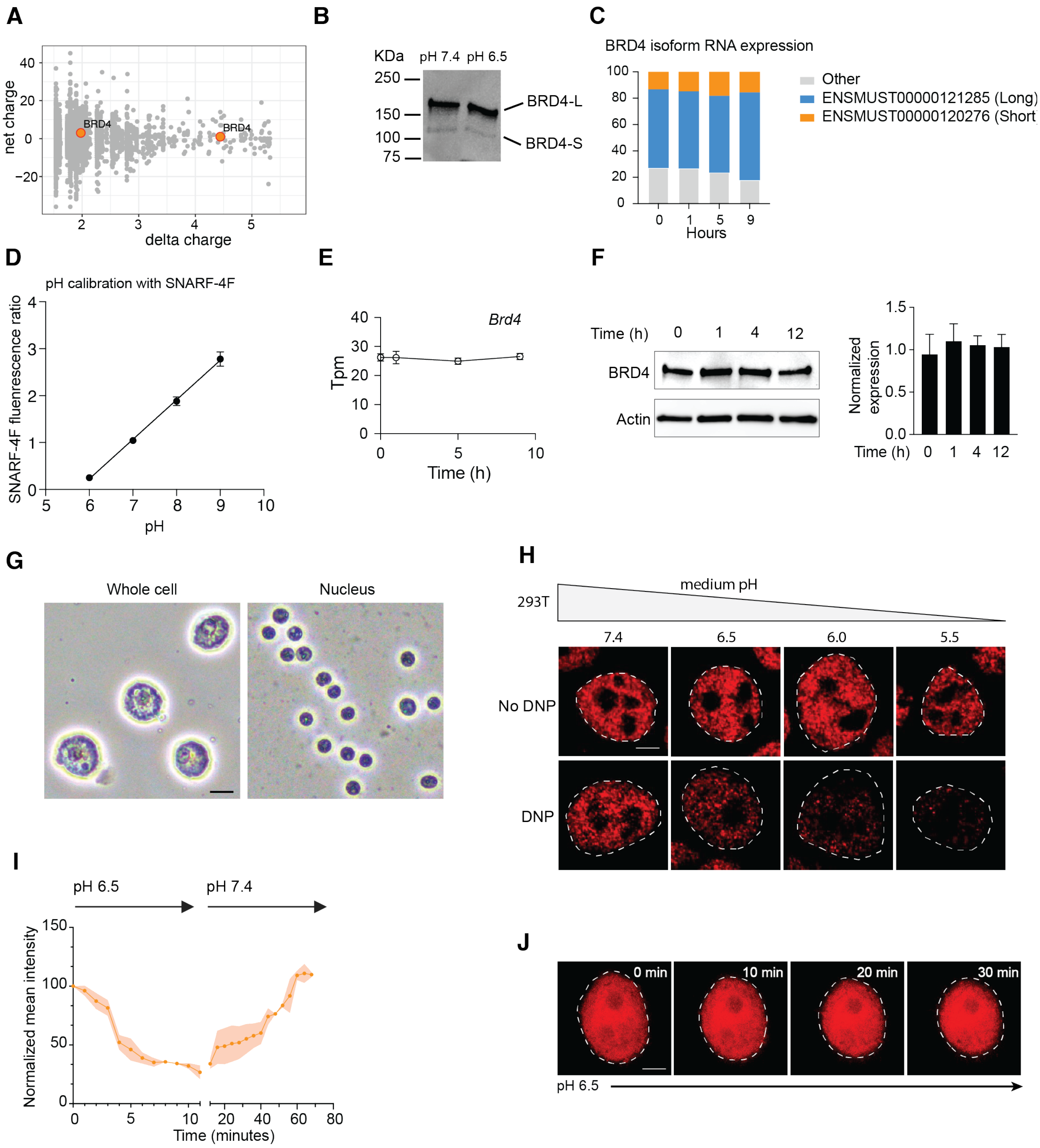

(A) Bioinformatic analysis of Δcharge and total charge of amino acid side chains for pH-sensitive peptide sequences shown in Figure 4A. **(B)** Western blot of BRD4 isoforms at pH 7.4 and pH 6.5. **(C)** Expression of various *Brd4* isoforms in murine BMDMs at pH 6.5. **(D)** Calibration curve for measuring pHi with SNARF-4F. R^2=0.999. **(E)** Gene expression of *Brd4* in BMDMs at acidic pH. **(F)** Western blot of BRD4 proteins in BMDMs under acidic pH for various time points. **(G)** Imaging of whole BMDM cells and isolated nuclei from BMDMs. **(H)** Immunofluorescent staining of BRD4 in 293T cells (E) conditioned at pH 7.4 - pH 5.5 for 4 hours, with or without 100 μM 2,3-DNP for 0.5 h. (**I**) Quantification of BRD4 in 293T cells for live cell imaging in Fig.4G. **(J)** Time lapse imaging of 293T cells stably expressing mCherry at acidic pH. Scale bar represents 2 μm.

bioRxiv preprint doi: https://doi.org/10.1101/2024.05.10.592432; this version posted May 30, 2025. The copyright holder for this preprint (which was not certified by peer review) is the author/funder, who has granted bioRxiv a license to display the preprint in perpetuity. It is made available under aCC-BY 4.0 International license.

Figure S5.

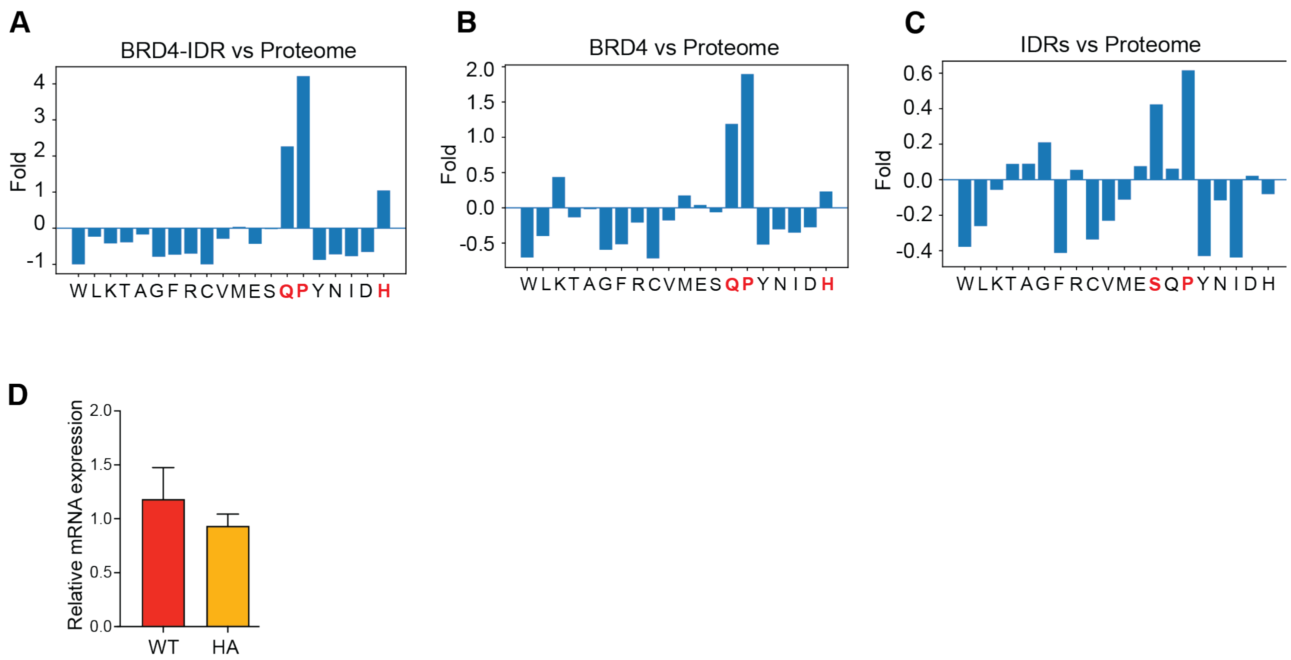

(**A-C**) Relative enrichment of amino acids between BRD4-IDR and the mouse proteome (A), BRD4 and the mouse proteome (B), and all IDRs and the mouse proteome (C). (**D**) Expression of exogenous BRD4 in $BRD4^{WT}$ (WT) and $BRD4^{HA}$ (HA) in 293T cells.

bioRxiv preprint doi: https://doi.org/10.1101/2024.05.10.592432; this version posted May 30, 2025. The copyright holder for this preprint (which was not certified by peer review) is the author/funder, who has granted bioRxiv a license to display the preprint in perpetuity. It is made available under aCC-BY 4.0 International license.

Figure S6.

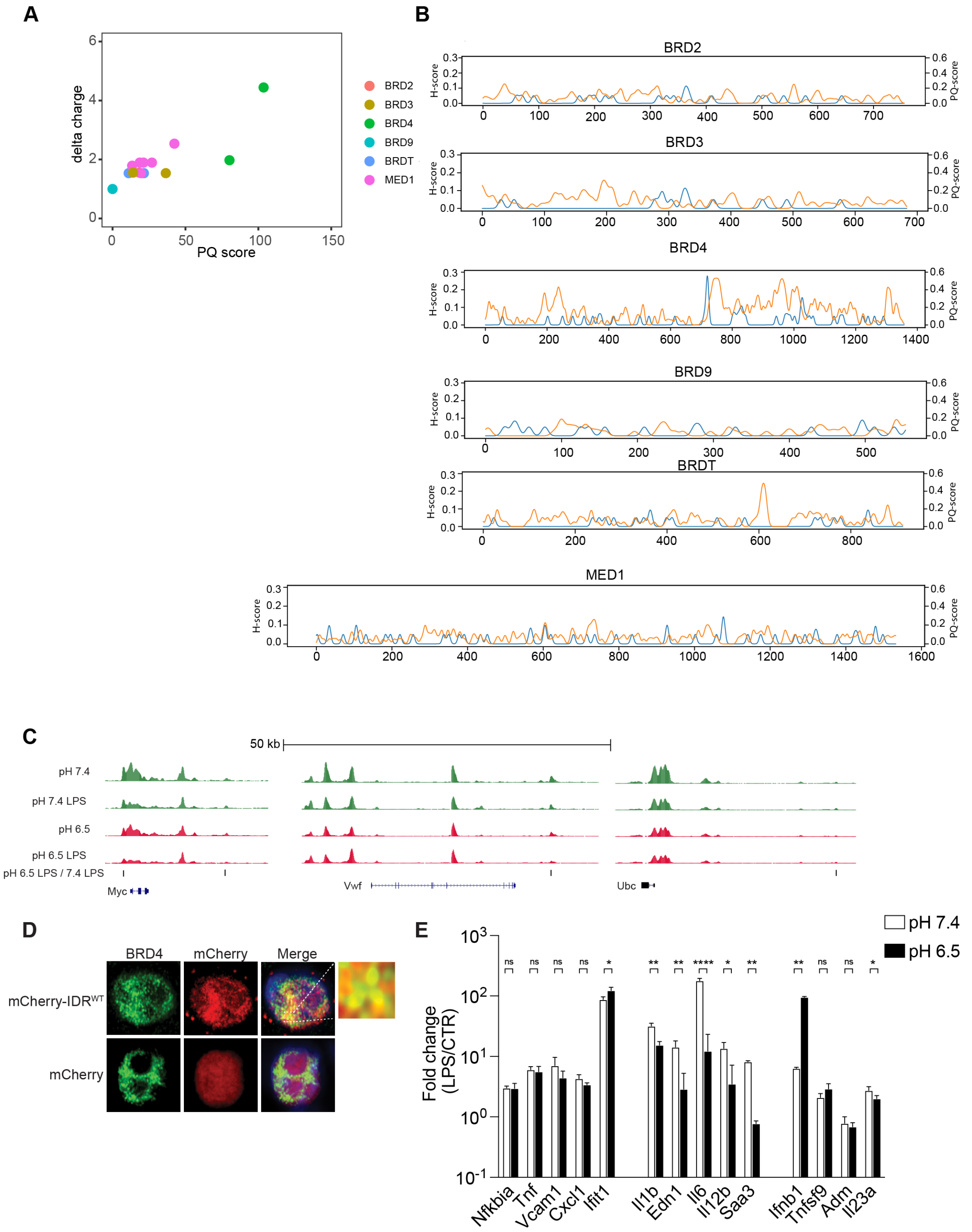

(**A**) Bioinformatic analysis of pH-sensitivity of BET family proteins and MED1. (**B**) Tracks of H, P and Q scores for full length BRD2, BRD3, BRD4, BRDT, BRD9 and MED1. (**C**) BRD4 ChIP-seq tracks at house-keeping genes *Myc*, *Vwf* and *Ubc*. Lines underneath the tracks mark the peaks with significant reduction of BRD4 occupancy comparing pH 6.5 LPS and pH 7.4 LPS. (**D**) Immunofluorescent imaging of mCherry fused BRD4-IDR (red), mCherry (red) and endogenous BRD4 (Green) in 293T cells. (**E**) Fold activation of inflammatory genes in iBMDMs after 6 h 100 ng/mL LPS at pH 7.4 or 6.5, normalized to unstimulated conditions respectively. Mean+/- standard deviation (STD). Unpaired t-test, Holm-Sidak's test for multiple comparisons.

bioRxiv preprint doi: https://doi.org/10.1101/2024.05.10.592432; this version posted May 30, 2025. The copyright holder for this preprint (which was not certified by peer review) is the author/funder, who has granted bioRxiv a license to display the preprint in perpetuity. It is made available under aCC-BY 4.0 International license.

Figure S7

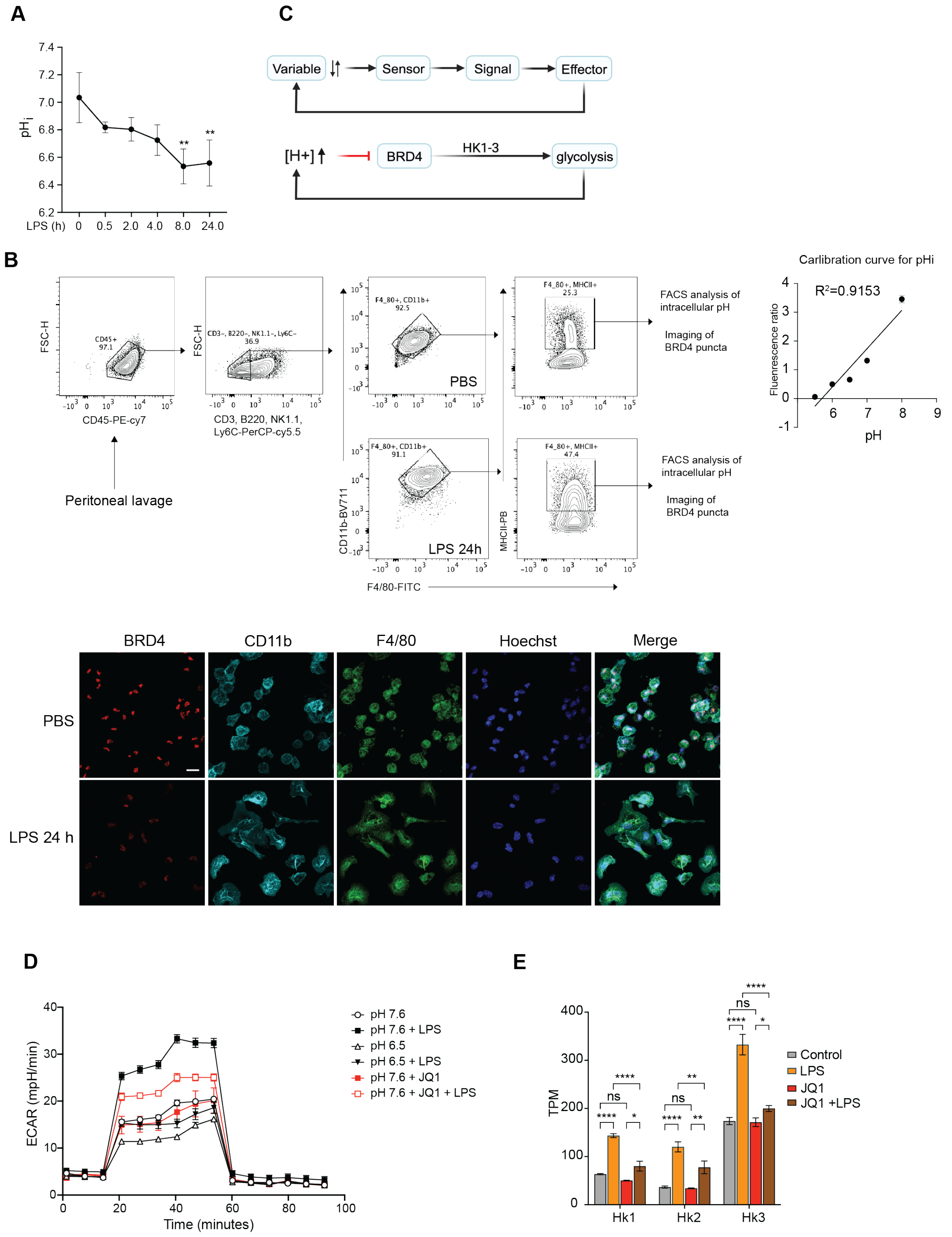

(**A**) pHi of BMDMs at various time points after 100 ng/mL LPS stimulation. ** p<0.01. (**B**) Flow cytometry gating strategy to isolate peritoneal macrophages, pH calibration, and immunofluorescent imaging of peritoneal macrophage staining 24 h after 3 mg/kg LPS treatment. (**C**) Diagram illustrating BRD4 as pH-sensor to control inflammatory response. (**D**) Seahorse analysis of ECAR on BMDMs treated with JQ1 or acidic pH. (**E**) The expression of *Hk1*, *Hk2* and *Hk3* with 100 ng/mL LPS or 0.5 μM JQ-1 treatment.

bioRxiv preprint doi: https://doi.org/10.1101/2024.05.10.592432; this version posted May 30, 2025. The copyright holder for this preprint (which was not certified by peer review) is the author/funder, who has granted bioRxiv a license to display the preprint in perpetuity. It is made available under aCC-BY 4.0 International license.

Supplemental movies

Supplemental movie 1: Live-cell time-lapse imaging of mCherry-BRD4 at pH 6.5 and recovery at pH 7.4

Supplemental movie 2: Live-cell time-lase imaging of mCherry-BRD4^{WT} at pH 6.5

Supplemental movie 3: Live-cell time-lase imaging of mCherry-BRD4^{HA} at pH 6.5

Supplemental tables

Supplemental table 1: qPCR primers used in this study

Supplemental table 2: Gene expression and expression components from deconvolution analysis.

The table includes both tpm and read count expression of BMDMs at pH 7.4, pH 7.4 LPS 4h, pH 6.5, pH 6.5 LPS 4h, fold changes, expression components (beta), covariance, p-values and R-square of the linear fitting, and gene cluster number.

Supplemental table 3: Bioinformatic screening of putative pH-sensitive proteins in the mouse proteome.
